# Engineering Enzyme‐Cleavable Oligonucleotides by Automated Solid‐Phase Incorporation of Cathepsin B Sensitive Dipeptide Linkers

**DOI:** 10.1002/ange.202114016

**Published:** 2022-02-10

**Authors:** Cheng Jin, Afaf H. EI‐Sagheer, Siqi Li, Katherine A. Vallis, Weihong Tan, Tom Brown

**Affiliations:** ^1^ Department of Chemistry, Chemistry Research Laboratory University of Oxford 12 Mansfield Road Oxford OX1 3TA UK; ^2^ The Cancer Hospital of the University of Chinese Academy of Sciences Zhejiang Cancer Hospital) Institute of Basic Medicine and Cancer (IBMC) Chinese Academy of Sciences Hangzhou Zhejiang 310022 China; ^3^ Institute of Molecular Medicine (IMM) Renji Hospital Shanghai Jiao Tong University School of Medicine Shanghai 200240 China; ^4^ Medical Research Council Oxford Institute for Radiation Oncology Department of Oncology University of Oxford Oxford OX3 7DQ UK; ^5^ Department of Science and Mathematics Suez University, Faculty of Petroleum and Mining Engineering Suez 43721 Egypt

**Keywords:** Cathepsin B, DNA Breakage, Dipeptide Linker, Oligonucleotide–Drug Conjugates, Solid-Phase Synthesis

## Abstract

Oligonucleotides containing cleavable linkers have emerged as versatile tools to achieve stimulus‐responsive and site‐specific cleavage of DNA. However, the limitations of previously reported cleavable linkers including photolabile and disulfide linkers have restricted their applications in vivo. Inspired by the cathepsin B‐sensitive dipeptide linkers in antibody–drug conjugates (ADCs) such as Adcetris, we have developed Val‐Ala‐02 and Val‐Ala‐Chalcone phosphoramidites for the automated synthesis of enzyme‐cleavable oligonucleotides. Cathepsin B digests Val‐Ala‐02 and Val‐Ala‐Chalcone linkers efficiently, enabling cleavage of oligonucleotides into two components or release of small‐molecule payloads. Based on the prior success of dipeptide linkers in ADCs, we believe that these dipeptide linker phosphoramidites will promote new clinical applications of therapeutic oligonucleotides.

## Graphical abstract

Cathepsin B‐sensitive dipeptide phosphoramidites were developed for automated synthesis of enzyme‐cleavable oligonucleotides. The enzymatic cleavage of Val‐Ala‐02 by cathepsin B is efficient in the context of single‐stranded DNA, but is sterically hindered by double‐stranded DNA and short hairpin loops. The design of Val‐Ala‐Chalcone allows the construction of oligonucleotide–drug conjugates which release their small‐molecule payload after enzymatic cleavage.

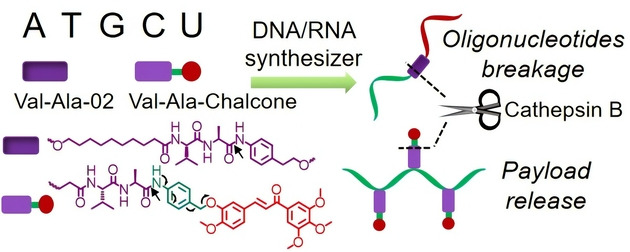

## Introduction

Cleavable linkers can be defined as molecules that covalently join two functional molecular entities through a cleavable bond.^[1]^ In chemical biology the molecular head groups serve to interact with, or manipulate, the biological target. Targeting molecules, small‐molecule drugs, fluorescence probes and nanoparticles have all been conjugated to cleavable linkers for drug development,^[2]^ proteomics studies^[3]^ and disease diagnostics.^[4]^ In the field of therapeutics there are examples of ADCs in which a chemotherapeutic drug is joined to an antibody by a cleavable covalent linker. By combining the antitumor activity of chemotherapeutic drugs and the tumor selectivity of antibodies, ADCs display a wider window for cancer chemotherapy than chemotherapeutic drugs alone.^[2a]^ ADCs with cathepsin B‐cleavable linkers are particularly effective, having high plasma stability. They are selectively cleaved in the lysosome of target tumor cells, thus releasing their payload. This clever design contributes greatly to the efficacy and safety of ADCs.^[5]^

In the nucleic acid field the incorporation of cleavable linkers into oligonucleotides enables the construction of stimuli‐responsive DNA that can undergo programmed site‐specific breakage. Cleavage of such oligonucleotides can trigger their conformational conversion, assembly/disassembly of nanostructures and drug release.^[6]^ Cleavable linkers which are sensitive to photoirradiation,^[7]^ enzymes,^[8]^ small molecules^[9]^ or ions^[10]^ have been developed for oligonucleotide‐based applications. Among these, the commercially available photocleavage (PC) and disulfide linkers are most commonly used. The PC linker is sensitive to ultraviolet photoirradiation and has the advantage of spatiotemporally controllable manipulation.^[11]^ Intracellular glutathione (GSH) can cleave the disulfide linker, facilitating biological applications of oligonucleotides that incorporate such linkages.^[12]^

**Figure 1:**
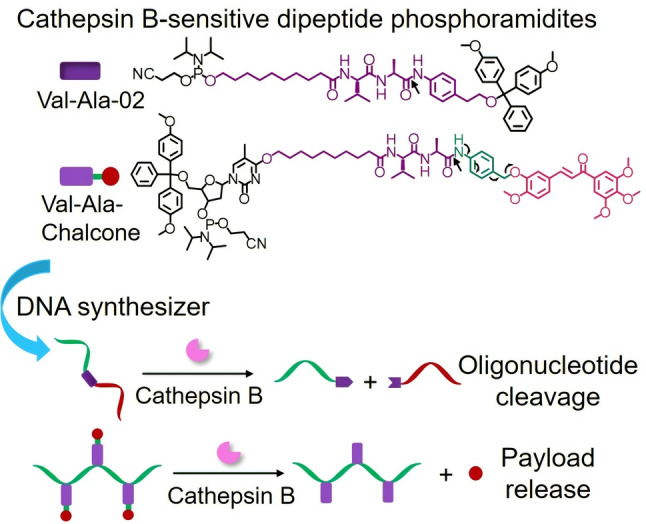
The automated synthesis and enzymatic cleavage of oligonucleotides incorporating Val‐Ala‐02 and Val‐Ala‐Chalcone linkers. The arrows in the chemical structures indicate the cleavage site of the dipeptide linker by cathepsin B and the cleavage mechanism.

In spite of these advances, both PC and disulfide linkers have limited in vivo applications and face challenges such as the phototoxicity and poor tissue penetration of ultraviolet photoirradiation and the limited stability of the disulfide linker during blood circulation. Herein, therefore, inspired by the cathepsin B‐cleavable dipeptide linkers in FDA‐approved ADCs (in particular Adcetris), we have developed dipeptide linker phosphoramidite monomers for the automated synthesis of enzyme‐cleavable oligonucleotides. We have synthesized two phosphoramidites, Val‐Ala‐02 and Val‐Ala‐Chalcone (Figure 1). Cathepsin B efficiently cuts oligonucleotides containing Val‐Ala‐02 into two pieces, and also cleaves Val‐Ala‐02‐incorporated hairpin and double‐stranded oligonucleotides provided that there is enough space between the peptide linker and the enzyme for the required molecular recognition. The design of Val‐Ala‐Chalcone allows the construction of oligonucleotide–drug conjugates which release their small‐molecule payload after enzymatic cleavage. Based on the prior success of cathepsin B‐cleavable linkers in ADCs, we believe that the dipeptide linkers reported in this work will inspire new clinical developments of therapeutic oligonucleotides.

## Results and Discussion

The key requirement for the automated incorporation of cathepsin B‐sensitive dipeptide linkers into oligonucleotides is the design and synthesis of the corresponding linker phosphoramidites. Guided by the structure of dipeptide linkers in ADCs,^[13]^ we initially synthesized Val‐Ala‐01 phosphoramidite in Figure S2a in which *p*‐aminobenzyl alcohol (PAB) is conjugated to the cleavable dipeptide moiety. Val‐Ala‐01 phosphoramidite can be incorporated with high efficiency during solid‐phase oligonucleotide synthesis, but the coupling yield of the monomer added directly after Val‐Ala‐01 decreases sharply, suggesting that Val‐Ala‐01 has poor stability in the synthesis cycle. The coupling efficiency by trityl analysis is shown in Figure S2b. After solid‐phase synthesis, mass spectrum analysis of the oligonucleotide containing Val‐Ala‐01 (Figure S2d) suggested a phosphite group at the terminus of the oligonucleotide, indicating that the PAB‐phosphate structure had degraded.

**Figure 2:**
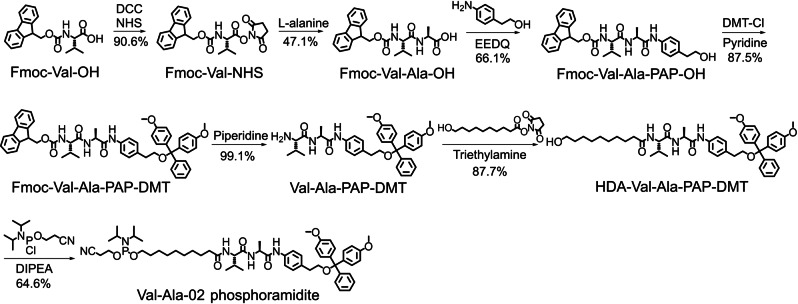
Synthesis of Val‐Ala‐02 phosphoramidite. DCC: N,N′‐dicyclohexylcarbodiimide. NHS: N‐hydroxysuccinimide. EEDQ: 2‐ethoxy‐1‐ethoxycarbonyl‐1,2‐dihydroquinoline. DMT‐Cl: 4,4′‐dimethoxytrityl chloride. DIPEA: N,N‐diisopropylethylamine.

Having confirmed the instability of the PAB‐phosphate structure during oligonucleotide synthesis, we then synthesized Val‐Ala‐02 phosphoramidite in which PAB is replaced with *p*‐aminophenethyl alcohol (PAP). As shown in Figure 2, Fmoc‐Val‐OH was activated with N‐hydroxysuccinimide (NHS) and N,N′‐dicyclohexylcarbodiimide (DCC), followed by reaction with L‐alanine to generate Fmoc‐Val‐Ala‐OH. Then, Fmoc‐Val‐Ala‐OH was reacted with PAP to produce Fmoc‐Val‐Ala‐PAP‐OH in 66.1 % yield. The hydroxy group of Fmoc‐Val‐Ala‐PAP‐OH was subsequently protected with the 4,4′‐dimethoxytrityl (DMT) group in 87.5 % yield, then Fmoc‐Val‐Ala‐PAP‐DMT was treated with 20 % piperidine in DMF to quantitatively remove the Fmoc group. The nucleophilic substitution reaction between 10‐hydroxydecanoic acid NHS ester (HDA‐NHS) and Val‐Ala‐PAP‐DMT provided HDA‐Val‐Ala‐PAP‐DMT as a solid foam. Finally, HDA‐Val‐Ala‐PAP‐DMT was converted into Val‐Ala‐02 phosphoramidite following a standard phosphitylation protocol. The product was characterized by ^1^H and ^31^P NMR (Figures S47 and S48), confirming the successful synthesis of Val‐Ala‐02 phosphoramidite.

Next, oligonucleotide ODN1 with a single Val‐Ala‐02 modification was assembled on an ABI394 DNA synthesizer. After synthesis, ODN1 was deprotected with a mixture of ammonium hydroxide and 40 % aqueous methylamine 1:1 v/v (AMA) for 30 minutes at 55 °C, followed by purification by high‐performance liquid chromatography (HPLC). The purified oligonucleotide was treated with 80 % aqueous acetic acid to remove the DMT group then characterized by mass spectrometry (Figure S21). This confirmed the successful synthesis of ODN1, and indicated that the Val‐Ala‐02 moiety is stable to oligonucleotide synthesis and subsequent deprotection.

**Figure 3:**
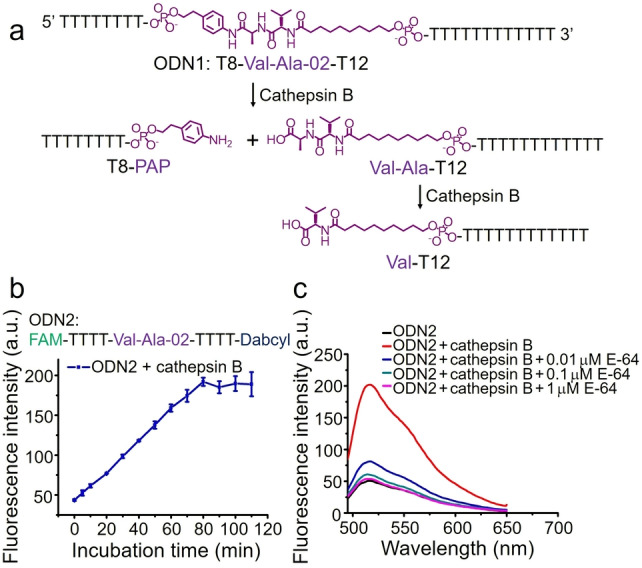
Cathepsin B‐mediated cleavage of oligonucleotides incorporating Val‐Ala‐02. a) Cathepsin B‐mediated cleavage reaction on ODN1. b) Fluorescence intensity of 2 μM ODN2 at 520 nm after incubation with 0.2 U mL^−1^ cathepsin B in buffer A for various incubation times. The error bars indicate the mean±SD values; *n*=3. c) Fluorescence spectra of 2 μM ODN2 treated with or without 0.2 U mL^−1^ cathepsin B in buffer A with or without E‐64 protease inhibitor (0.01 μM, 0.1 μM or 1 μM). The incubation time is one hour.

We then investigated the enzymatic cleavage of ODN1 by cathepsin B. ODN1 was dissolved in 25 mM sodium acetate, 5 mM dithiothreitol (DTT), at pH 5.0 (buffer A) to a concentration of 5 μM and incubated with 0.2 U mL^−1^ cathepsin B at 37 °C for various time intervals (Table S4). The oligonucleotides were then separated by HPLC and analyzed by mass spectrometry. As shown in Figure S3, ODN1 shows excellent stability in buffer A over 1 hour (entry 1 in Table S4), and after incubation with cathepsin B for 1 hour (entry 2 in Table S4), ODN1 disappeared and two new DNA peaks emerged. The molecular weights of the two newly generated DNA peaks are consistent with T8‐PAP and Val‐Ala‐T12, respectively (Figure S4). This indicates that 0.2 U mL^−1^ cathepsin B had completely cut ODN1 into T8‐PAP and Val‐Ala‐T12 (Figure 3a). To determine if the resultant T8‐PAP or Val‐Ala‐T12 could be further cleaved after longer incubation times, ODN1 was incubated with cathepsin B for six and twenty‐two hours. Most of the Val‐Ala‐T12 was converted into Val‐T12 after six hours of incubation (Figure S5) and the conversion was essentially complete after twenty‐two hours (Figure S6). However, T8‐PAP showed no obvious change after six hours (Figure S5). Therefore, we propose the cathepsin B‐mediated cleavage reaction of ODN1 in Figure 3a in which cathepsin B first cleaves ODN1 into T8‐PAP and Val‐Ala‐T12, and Val‐Ala‐T12 is further converted to Val‐T12.

To monitor enzymatic cleavage of Val‐Ala‐02 in real time, ODN2 was dual‐labeled with a fluorophore and quencher at the termini (Figure 3b). The fluorescence of intact ODN2 is quenched, but its fluorescence is expected to recover when Val‐Ala‐02 cleaved due to separation of fluorophore and quencher. To confirm the hypothesis, ODN2 (2 μM) was incubated with 0.2 U mL^−1^ cathepsin B in buffer A at 37 °C for various times and the fluorescence spectra were recorded. As shown in Figures 3b and S7, almost all Val‐Ala‐02 in ODN2 was cleaved by cathepsin B within 80 minutes. E‐64, a commercial protease inhibitor, was used to inhibit the activity of cathepsin B to provide additional proof that cathepsin B is responsible for the enzymatic cleavage of Val‐Ala‐02. As expected, cathepsin B failed to cleave Val‐Ala‐02 in ODN2 in buffer A containing 0.1 μM of E‐64 (Figure 3c), demonstrating again that cathepsin B induces the enzymatic cleavage of Val‐Ala‐02 in linear single‐stranded oligonucleotides.

**Figure 4:**
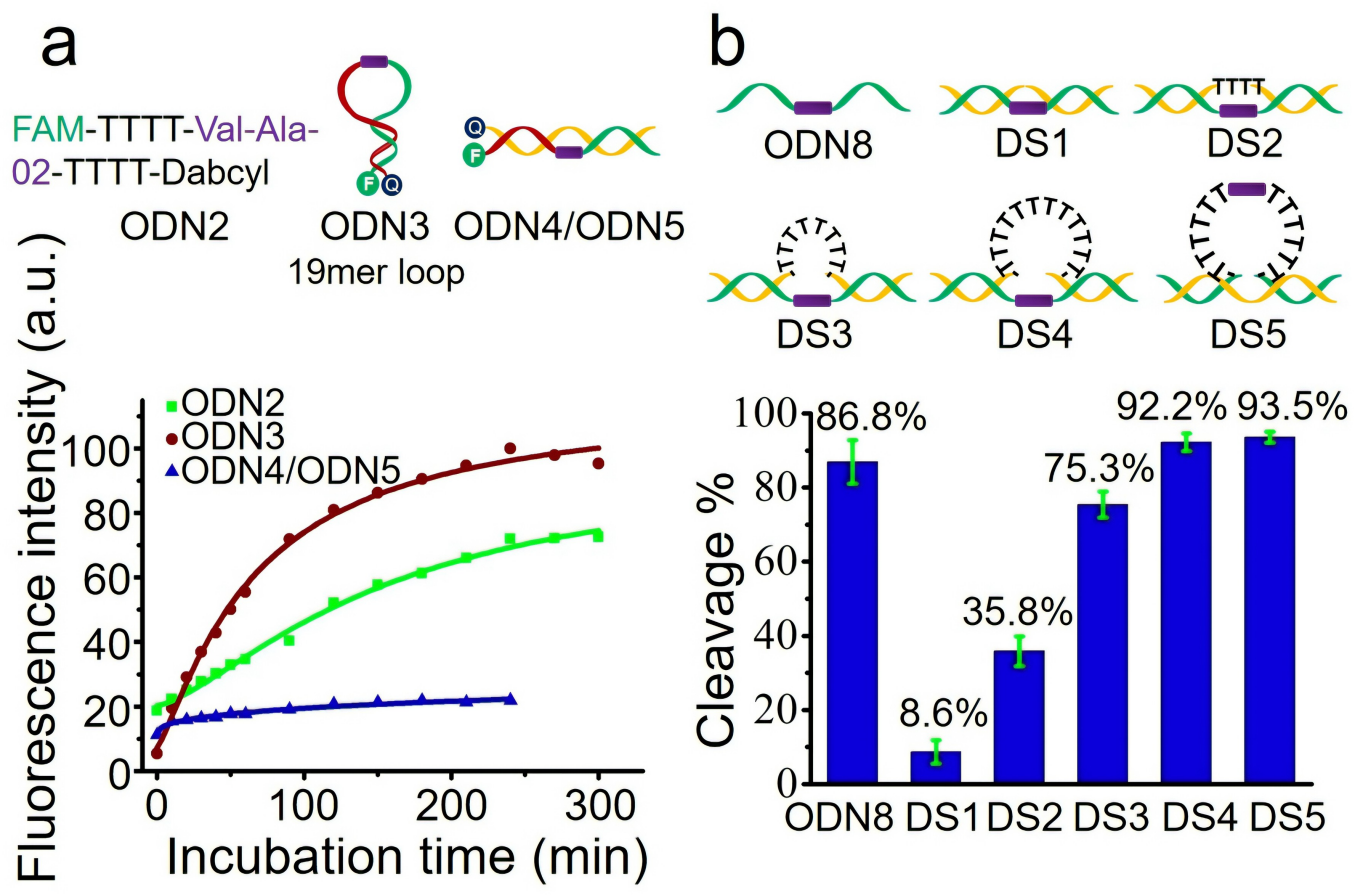
Cathepsin B‐induced cleavage of Val‐Ala‐02 in various DNA structures. a) Fluorescence intensity of 1 μM single‐stranded ODN2, hairpin ODN3 and double‐stranded ODN4/ODN5 oligonucleotides incorporating Val‐Ala‐02. Fluorescence was measured at 520 nm after incubation with 0.1 U mL^−1^ cathepsin B in buffer B for various times. b) Enzymatic cleavage of Val‐Ala‐02 in double‐stranded oligonucleotides with various loop structures after one hour of incubation with 0.5 U mL^−1^ cathepsin B in buffer B. The error bars indicate the mean±SD values; *n*=3.

It is well known that molecular recognition between the active site of an enzyme and substrate requires complementarity in terms of shape and other properties. As the cathepsin B protein is a large molecule (339 amino acids), it is likely that steric hindrance will interfere with its interactions with certain DNA structures.^[14]^ Oligonucleotides can assemble into many secondary and tertiary structures, such as, hairpins, duplexes, quadruplexes in a sequence‐dependent manner, and these structures are sterically crowded and rigid compared to unstructured single strands. Therefore, we investigated the cathepsin B‐responsive cleavage of Val‐Ala‐02 in hairpin and double‐stranded structures to determine if steric hindrance can affect the interaction between Val‐Ala‐02 and cathepsin B. Linear single‐stranded (ODN2), hairpin (ODN3) and linear double‐stranded (ODN4/ODN5) oligonucleotides (1 μM) were incubated with 0.1 U mL^−1^ cathepsin B in buffer B (25 mM sodium acetate, 5 mM DTT, 1 mM MgCl_2_, pH 5.0) at 37 °C for various times, and fluorescence spectra were recorded. As shown in Figure 4a, both ODN2 and ODN3 were completely cleaved within four hours. However, no obvious change in fluorescence intensity was observed for the ODN4/ODN5 duplex, suggesting that cathepsin B can barely cut Val‐Ala‐02 within a double‐stranded structure. Having found that the hairpin oligonucleotide ODN3 with a long loop can be cut efficiently, we studied the enzymatic cleavage of Val‐Ala‐02 in hairpin ODN6 with a very short 2mer loop region. As shown in Figure S11, compared to ODN3, ODN6 shows significantly slower cleavage, demonstrating that hairpins with shorter loop structures, i.e., less space for enzymatic recognition of Val‐Ala‐02, are more difficult to cleave. As well as hairpin ODN6, we also investigated the enzymatic cleavage of Val‐Ala‐02 in hairpin ODN7 with a 6mer loop region. As shown in Figure S12, only 16.5 % and 40.4 % Val‐Ala‐02 in ODN7 were cleaved by 0.5 U mL^−1^ cathepsin B after one and three hours, respectively. These results confirm that the cathepsin B‐responsive cleavage of Val‐Ala‐02‐incorporated oligonucleotides is sensitive to steric factors in structured DNA.

To further explore the sensitivity of cleavage of Val‐Ala‐02 by cathepsin B to steric factors, a series of double‐stranded oligonucleotides with various loop sizes in their complementary DNA (cDNA) were designed. As shown in Figures 4b and S13, the series of oligonucleotides DS1, DS2, DS3 and DS4 have zero, four, eight and twelve unpaired thymine bases respectively opposite Val‐Ala‐02. After one hour of incubation, the percentage of enzymatic cleavage in DS1, DS2, DS3 and DS4 was 8.6 %, 35.8 %, 75.3 % and 92.2 %, respectively. Hence the larger the loop the more efficient the enzymatic cleavage. Next, DS5, a double‐stranded oligonucleotide in which Val‐Ala‐02 was incorporated symmetrically within a 12mer loop instead of in the strand opposite to the loop, was designed. The strand containing the dipeptide was 93.5 % cleaved by cathepsin B after one hour of incubation (Figures 4b and S14). This result indicates that the 12mer loop shows negligible steric hindrance against enzymatic cleavage of Val‐Ala‐02 if the dipeptide is incorporated in the middle of loop region.

The marked difference in the enzymatic cleavage rates of Val‐Ala‐02 in linear single‐stranded and double‐stranded DNA is an indication that the cleavage of Val‐Ala‐02 might be controlled by conversion between single‐stranded and double‐stranded structures containing the dipeptide. Carrying this idea forward, we reasoned that toehold‐mediated strand displacement could be a powerful molecular tool to achieve controlled DNA cleavage by exchanging one strand for another. Therefore, a displacement strand (ODN15) was used to hybridize with ODN14, thereby releasing ODN4 from ODN4/ODN14. In a control reaction the enzymatic cleavage of the Val‐Ala‐02 dipeptide in single stranded ODN4 by cathepsin B after one hour of incubation was 75 % (lane 2, Figure S15b). In contrast, 9.9 % of Val‐Ala‐02 in the ODN4/ODN14 duplex was cleaved by cathepsin B under the same conditions (lane 5, Figure S15b). However, the addition of ODN15 released ODN4 from the ODN4/ODN14 duplex, and consequently 62.9 % of ODN4 was cleaved in 1 hour and 82 % in 3 hours (lanes 8 and 9, Figure S15b). This cumulative evidence demonstrates that the cathepsin B‐responsive cleavage of Val‐Ala‐02 can be controlled by the formation or dissociation of a specific double‐stranded structure in which recognition of Val‐Ala‐02 by cathepsin B is affected by steric hindrance.

The stability of Val‐Ala‐02 in biological fluids is critical to its biomedical applications. Accordingly, the stability of Val‐Ala‐02 in fetal bovine serum (FBS) was investigated. To improve the resistance of the oligonucleotide against enzymatic degradation, stabilized 2′‐OMe−U (U_OMe_) nucleotides were incorporated. As shown in Figure S16c, FAM and Dabcyl dual‐labeled ODN16 exhibits a slow increase of fluorescence intensity after incubation with 20 % FBS in Dulbecco's phosphate‐buffered saline (DPBS). However, compared to ODN16, oligonucleotide ODN17, without Val‐Ala‐02, shows a slightly increased fluorescence intensity (Figure S16e), indicating that Val‐Ala‐02 exhibits good stability in 20 % FBS solution, at least better than the stabilized U_OMe_ nucleic acid strand. In addition, no obvious decline of cell viability of HepG2 cells was observed after treatment with ODN1 for 72 hours, indicating that Val‐Ala‐02 shows negligible cell cytotoxicity, even at a concentration of up to 20 μM (Figure S17). These results show the potential for future in vivo applications of oligonucleotides incorporating Val‐Ala‐02.

Cathepsin B is a cysteine proteases that localizes largely in endosomes and lysosomes where oligonucleotides also tend to accumulate. Importantly, therapeutic oligonucleotides are stable in this environment due to modifications to their sugar‐phosphate backbone.^[15]^ An extreme example is the clinically approved drug inclisiran, (Leqvio, Novartis) which only requires administration at six‐monthly intervals. Taking this into account modified oligonucleotides containing cleavable Val‐Ala‐02 linkers are realistic candidates for in vivo applications.

In addition to its incorporation into oligonucleotides as an enzyme‐cleavable linker for DNA strand scission, the dipeptide linker can be conjugated with small‐molecule drugs for the construction of oligonucleotide–drug conjugates. Automated attachment of small‐molecule drugs to oligonucleotides through phosphoramidite chemistry and solid‐phase oligonucleotide synthesis has emerged as a powerful technology in constructing payload‐tunable and structure‐defined oligonucleotide–drug conjugates.^[16]^ As such, many chemotherapeutic drug phosphoramidites, e.g., floxuridine,[^16^, ^17^] combretastatin A‐4^[18]^ and 5‐fluorouracil,^[6d]^ have been conjugated to oligonucleotides for targeted cancer chemotherapy. We therefore decided to develop a dipeptide‐drug phosphoramidite for the automated synthesis of oligonucleotide–drug conjugates with programmed cathepsin B‐mediated drug release.

Since Val‐Ala‐01 is unstable during oligonucleotide synthesis, we decided to design a new monomer to allow us to directly conjugate small molecules with PAB without using a phosphodiester linkage. To achieve this, Fmoc‐Val‐Ala‐PAB‐OH was brominated by N‐bromosuccinimide (NBS) and triphenylphosphine (PPh_3_). The resultant Fmoc‐Val‐Ala‐PAB‐Br was reacted with 7‐hydroxycoumarin and K_2_CO_3_ in DMF solution. The Fmoc group was deprotected, and Val‐Ala‐PAB‐Coumarin was obtained as a light‐yellow solid in 47.3 % yield. The nucleophilic substitution reaction between HDA‐NHS and Val‐Ala‐PAB‐Coumarin provided HDA‐Val‐Ala‐PAB‐Coumarin in 67.6 % yield. Finally, HDA‐Val‐Ala‐PAB‐Coumarin was converted into Val‐Ala‐Coumarin phosphoramidite for use in oligonucleotide synthesis (Figure S34). To evaluate the monomer, we synthesized ODN18 which was deprotected by treatment with 50 mM K_2_CO_3_ in methanol at room temperature for three hours, then purified by HPLC. The success of the modified oligonucleotide synthesis was confirmed by mass spectrometry (Figure S31).

**Figure 5:**
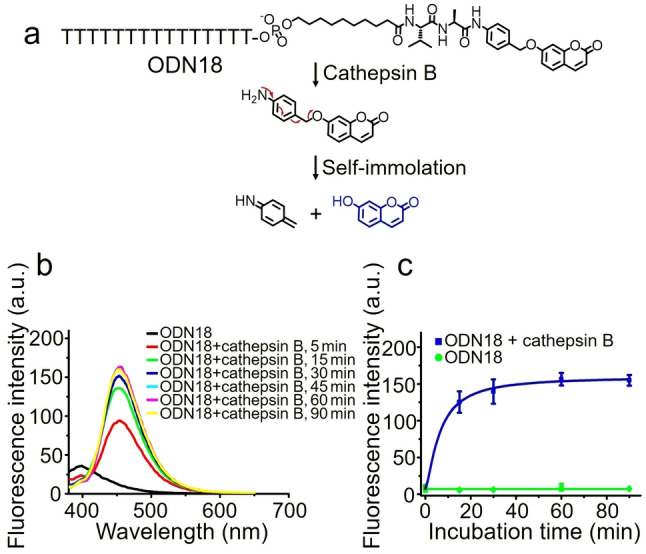
Cathepsin B‐mediated cleavage of ODN18. a) Cleavage of ODN18 and spontaneous release of 7‐hydroxycoumarin. b) Fluorescence spectra of 1 μM ODN18 before and after incubation with 0.5 U mL^−1^ cathepsin B in buffer A at 37 °C for various times. c) Comparison of fluorescence intensity of 1 μM ODN18 at 454 nm with (blue line) and without (green line) treatment with 0.5 U mL^−1^ cathepsin B in buffer A at 37 °C. The error bars indicate the mean±SD values; *n*=3.

7‐Hydroxycoumarin is a fluorescent dye with bright emission at 454 nm, but its fluorescence emission is strongly suppressed if the hydroxyl group is protected as an ether.^[19]^ Therefore, the enzymatic cleavage‐induced‐release of 7‐hydroxycoumarin from ODN18 can be monitored by a change in fluorescence (Figure 5a). As shown in Figure 5b, ODN18 shows a fluorescence emission peak at 400 nm. However, after the addition of cathepsin B, this peak disappears; instead, a new fluorescence emission peak emerges at 454 nm, caused by the spontaneous release of 7‐hydroxycoumarin from ODN18 after enzymatic cleavage. In contrast, without cathepsin B, ODN18 shows a negligible change of fluorescence intensity at 454 nm, even with incubation times up to 90 minutes (Figure 5c). These findings demonstrate that cathepsin B can cleave Val‐Ala‐Coumarin to release small‐molecule payloads.

**Figure 6:**
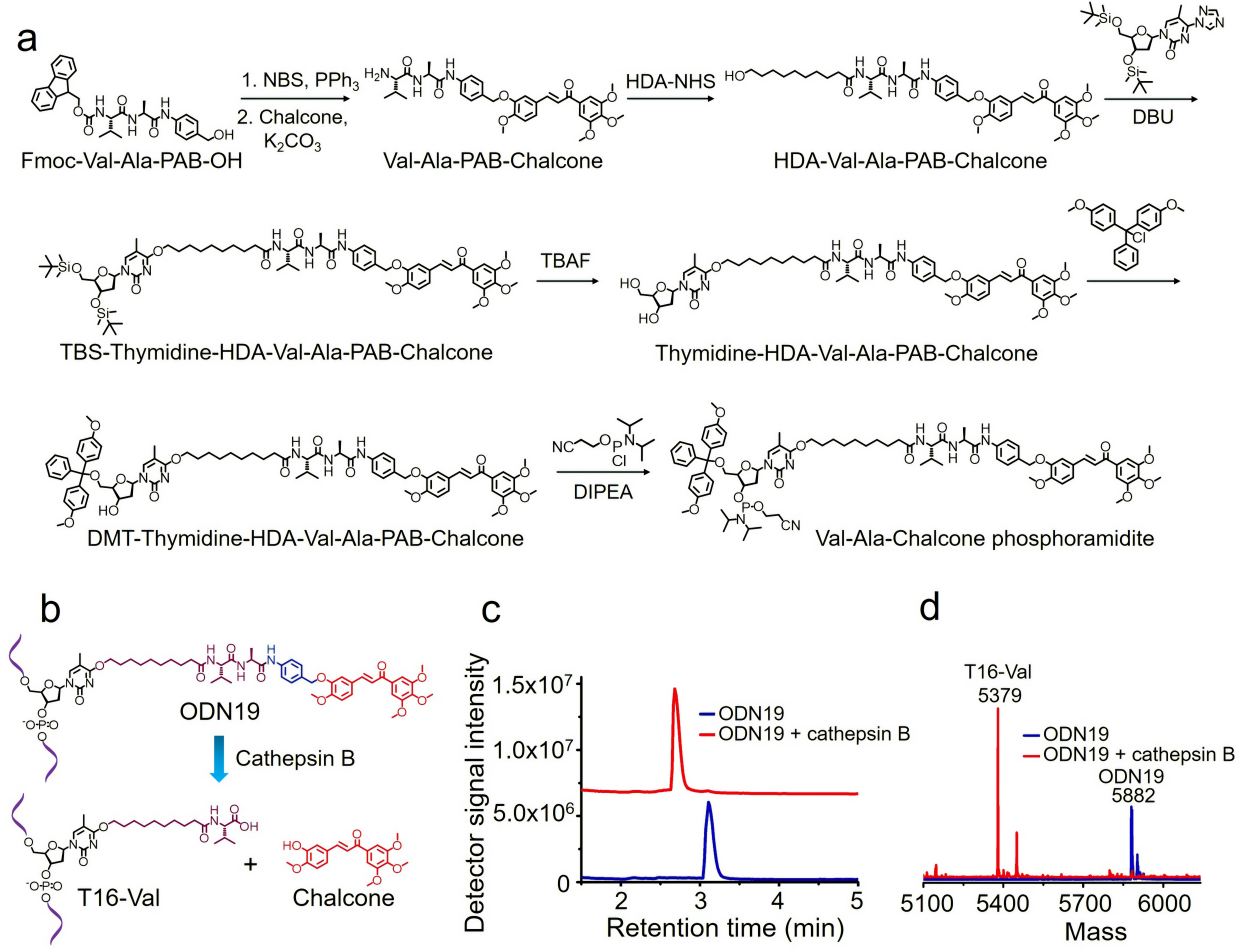
Synthesis of Val‐Ala‐Chalcone phosphoramidite and cathepsin B‐mediated cleavage of ODN19. a) Synthesis of Val‐Ala‐Chalcone phosphoramidite. NBS: N‐bromosuccinimide. PPh_3_: triphenylphosphine. DBU: 1.8‐diazabicyclo[5.4.0]undec‐7‐ene. TBAF: tetrabutylammonium fluoride. DMT‐Cl: 4,4′‐dimethoxytrityl chloride. DIPEA: N,N‐diisopropylethylamine. b) Cathepsin B‐mediated cleavage of ODN19 and the release of chalcone. c) HPLC chromatograms of 10 μM ODN19 after incubation with (red line) or without (blue line) 0.2 U mL^−1^ cathepsin B in buffer A at 37 °C for four hours. d) Mass spectra of ODN19 after incubated with (red line) and without (blue line) 0.2 U mL^−1^ cathepsin B in buffer A at 37 °C for four hours. After enzymatic cleavage, ODN19 (5880.3 Da) was converted to T16‐Val (5377.7 Da).

Encouraged by the enzymatic cleavage‐induced release of 7‐hydroxycoumarin from ODN18, we extended this idea to develop a dipeptide‐drug phosphoramidite for use in the automated synthesis of oligonucleotide–drug conjugates. A chalcone derivative that is able to inhibit cellular microtubule polymerization was employed as a working example (Figure S20).^[20]^ Val‐Ala‐Chalcone phosphoramidite was developed for this purpose (Figure 6a), and an oligonucleotide with the Val‐Ala‐Chalcone modification (ODN19) was synthesized (Table S1). After synthesis, ODN19 was characterized by mass spectrometry, proving its successful synthesis (Figure S32).

We investigated the enzymatic cleavage of ODN19 by cathepsin B. ODN19 was diluted with buffer A to a final concentration of 10 μM and then incubated with 0.2 U mL^−1^ cathepsin B at 37 °C for four hours. When the incubation was complete, the oligonucleotide was purified by HPLC and analyzed by mass spectrometry. As shown in Figures 6c and d, the retention time of uncleaved ODN19 is 3.11 minutes and its molecular weight is 5882 Da. After enzymatic cleavage, the oligonucleotide peak for ODN19 at 3.11 minutes disappeared; instead, a new oligonucleotide peak at 2.68 minutes was observed with a molecular weight of 5379 Da, consistent with the molecular weight of T16‐Val (5377.7 Da). Hence cathepsin B is able to efficiently cleave Val‐Ala‐Chalcone in ODN19 and thus release chalcone. Finally, for in vivo applications the Val‐Ala‐Chalcone linker must be stable in serum. To confirm this we incubated HDA‐Val‐Ala‐PAB‐Chalcone in 95 % FBS for up to 24 hours. In these extreme conditions the molecule was not degraded (Figures S18, S19).

## Conclusion

We have developed Val‐Ala‐02 and Val‐Ala‐Chalcone phosphoramidites for the synthesis of cathepsin B‐cleavable oligonucleotides and oligonucleotide–drug conjugates. Both these cleavable dipeptide linkers can be efficiently incorporated into oligonucleotides via automated DNA synthesis to produce constructs with well‐defined molecular structures and tunable payloads. The molecular recognition and cleavage of Val‐Ala‐02 by cathepsin B is efficient in the context of single‐stranded DNA, but is sterically hindered by double‐stranded DNA and short hairpin loops. However, cathepsin B efficiently cleaves Val‐Ala‐02 in double‐stranded oligonucleotides if a single‐stranded loop of sufficient length is present in the complementary DNA strand opposite Val‐Ala‐02. Taking these design criteria into account, Val‐Ala‐02 could be used in the cathepsin B‐responsive release of oligonucleotides from nanomaterials (e.g. nanoconstructs designed to enhance intracellular delivery) for gene therapy and other therapeutic applications. We have also shown that Val‐Ala‐Chalcone can potentially be used to develop oligonucleotide–drug conjugates with applications in targeted cancer chemotherapy, for example in aptamer–drug conjugates (ApDC). In addition to our own serum data (Figure S19) numerous studies have shown that cathepsin B‐sensitive dipeptide linkers have excellent stability during circulation in blood. Hence, given that cathepsin B is overexpressed in several human cancers, and may be related to tumorigenesis,^[21]^ oligonucleotides incorporating dipeptide linkers of the kind developed in this study may have applications as molecular probes for disease diagnosis and cancer therapy.

## Conflict of interest

The authors declare no competing financial interest.

## Supporting information

As a service to our authors and readers, this journal provides supporting information supplied by the authors. Such materials are peer reviewed and may be re‐organized for online delivery, but are not copy‐edited or typeset. Technical support issues arising from supporting information (other than missing files) should be addressed to the authors.

Supporting Information
